# Chronic energy deficiency and associated factors among older population in Ethiopia: A community based study

**DOI:** 10.1371/journal.pone.0214861

**Published:** 2019-04-10

**Authors:** Mulatu Legesse, Zegeye Abebe, Haile Woldie

**Affiliations:** 1 Department of Pharmacology, Dean Office of Students Services, College of St. Fasil, the University of Gondar, Gondar, Ethiopia; 2 Department of Human Nutrition, Institute of Public Health, College of Medicine and Health Sciences, the University of Gondar, Gondar, Ethiopia; University of Malaya, MALAYSIA

## Abstract

**Introduction:**

Chronic energy deficiency is an important public health problem among older (aged ≥ 65 years) population. Globally, one in seven older people has a medium to high risk of malnutrition. The situation of chronic energy deficiency among older people is quite poorly known in Ethiopia. Therefore, this study was aimed to determine the magnitude of chronic energy deficiency and associated factors among elders’ aged ≥ 65 years, in Aykel town administration, Amhara Regional State, Northwest Ethiopia.

**Methods:**

A community based cross-sectional survey was carried out from March 28^th^ to April 20^th^, 2018. Study participants were recruited by a census technique. Both bivariate and multivariate logistic regression analysis used to identify factors associated with chronic energy deficiency. All variables with p–values of < 0.2 in the bivariate analysis were remarked for the multivariable analysis. Both Crude Odds Ratio (COR) and Adjusted Odds Ratio (AOR) were computed to determine the strength of association. In the multivariate analysis, all variables at p–values of < 0.05 were considered as statistically significant.

**Results:**

A total of 892 participants recruited for the study. The prevalence of chronic energy deficiency was 17.6% (95%CI: 15.00, 20.20) among the study community. It was significantly associated with female sex (AOR: 1.58; 95%CI: 1.04, 2.41), age (AOR: 3.90; 95%CI: 1.85, 8.25), household food insecurity (AOR: 1.95; 95%CI: 1.16, 3.00), poor household wealth status (AOR: 1.77; 95%CI: 1.07, 2.94), loss of appetite due to illness (AOR: 2.93, 95%CI: 1.92, 4.48) and poor dietary diversity score (AOR: 5.51; 95%CI: 2.89, 10.52).

**Conclusions:**

The magnitude of chronic energy deficiency was high in the study area. It was significantly associated with female sex, age, poor dietary diversity score, loss of appetite due to illness, household food insecurity and poor wealth status. Therefore, there is a need to design and implement programs and strategies to improve nutritional status particularly focusing on female older population in improving dietary practices and food security. In addition, improving household economic and living standards is an essential measure to address the burden of CED among the older community.

## Introduction

The older population is growing worldwide in both developed and developing countries. It is projected to reach 2 billion by 2050, with the highest increase in developing countries [1, 2]. The issue of population aging in developing countries entails more serious problems than in developed countries, as the increase is a relatively recent phenomenon and those countries are not yet prepared to manage [3].

Older population are also subjected to many health and nutrition related outcomes [4], including reduced cognition, loss of lean body and skeletal mass, inflammatory stress, compromised immune function, susceptibility to infection, impaired physical function, chronic energy deficiency (CED), depression, increased dependence, and reduced quality of life [5, 6].

Chronic energy deficiency (Body mass index less than 18.5kg/m^2^) is a major public health issue among elders aged ≥ 65 years throughout the world. It is estimated that one in seven people aged ≥ 65 years has a medium to high risks of CED. The problem is substantial and persistent in the continent of Africa. Especially, in the Sub Sahara African region, it ranges from 6–48% among older population. Similarly, it is a significant public health problem in Ethiopia, the problem ranges from 21.9–28.3% among older population [7–9].

The causes of CED among elders is a multi-factorial. Hence, studies have been documented that socio-demographic and economic characteristics, such as residence, sex, age, marital status, level of education, household economic and food insecurity status [10–12]; co-morbidity and health care service related factors, such as subsequent hospitalization, level of self-perception, medication use, chronic and acute infections and functional impairment of the body [13, 14]; and factors related to dietary and feeding practices, including dietary diversity scores, meal frequency, source of food for the household, time to have meal and ability to self-feeding were the determinants of CED among older population [13, 15].

The government of Ethiopia has launched many programmes and strategies to reduce the burden of malnutrition for different segments of its population [16, 17]. Surprisingly, no government policy exists in Ethiopia to addresses the health and nutritional problems among the older population. However, the country is facing daunting both demographic growth and population ageing. It is a time to pursue policies that jointly address the rapid population ageing of the country and its health and nutritional problems.

The rights of older population to basic needs of food, health and nutritional care to be actively protected and promoted in Ethiopia. Conversely, the current situation of older people in Ethiopia is quite poorly known, and studies are scarce. Therefore, understanding the cause of CED among older people has utmost importance to arrest the downward spiral of the problem. Furthermore, it is vital to step to design important interventions to improve the overall health and nutritional wellbeing of older population. Hence, this study was carried out to determine the magnitude and determinant factors of CED among people aged ≥ 65 years in Aykel town administration, Northwest Ethiopia.

## Methods

### Study design, setting and period

A community-based cross-sectional survey was carried out from March 28^th^ to April 20^th^ 2018 in Aykel town administration, Amhara regional state, Northwest Ethiopia. The study area is located approximately 761 km far from the capital city of Ethiopia, Addis Ababa. Aykel town is found at a latitude and longitude of 12°31′ 0"N 37°4′60"E with an elevation of 2100 meters above the sea level. Based on the 2017/2018 population projection Central Statistical Agency of Ethiopia; Aykel town administration had a total population of 53,581 (25,175 and 28,406 females). A total of 921 people were aged ≥ 65 years [18]. The town administration has two kebeles (01 and 02) with a population number of 28,489 and 25,092 respectively [18]. Furthermore, there are one primary hospital, two governmental health centers and six private clinics found in the study area.

### Sample size determination, sampling technique and study population

The required sample size for the study was estimated by a single population proportion formula using the following assumptions: 95% confidence interval, 3% of margin of error and a proportion of 21.9% CED among older people aged ≥ 65 years [7]. Finally, the size of 803 was obtained by considering a 10% non-response rate. However, as a result of small the number of the source population (N = 921), the survey was conducted by including the whole people aged ≥ 65 years found in the study area.

### Data collection tools and procedures of the study

An interviewer administered, pre-tested and structured questionnaire was used to collect the data. The questionnaire was constructed into three major parts, such as socio-demographic and economic related factors, co-morbidity and health care related factors, and dietary and feeding practices related characteristics. A total of five data collectors (four nurses as a data collector and one Public Health Officer as a supervisor) were recruited. The dietary diversity scores (DDSs) of participants were measured using the 24-hours recall method. A recall period in the past 24 hours of having a meal is the mostly chosen method because of its low subjects to recall error, less bulky for respondents, and conforms to the recall period. In addition, the tool uses an open recall method to collect data about all foods and drinks taken by participants in the previous 24-hours of an interview. Participants were requested to list what food groups were consumed in the past 24 hours of the survey. The nine food groups were used to compute the DDSs of study subjects. It comprises; grains, roots, and tubers; legumes and nuts; dairy products; flesh foods (meat, fish, poultry and organ meats); eggs; vitamin A rich fruits and vegetables; other fruits and vegetables. Finally, the score was defined as; “Poor” (when the DDS is ≤ 3), “Medium” (when the DDS is betwwen 4–5) and “High” (when the DDS is ≥ 6) [19].

Household food security status (HFSS) of participants was measured by using the FANTA Household Food Insecurity Access Scale. It is specifically designed to measure household limited access to food in the previous one month. The tool is constructed from nine consecutive food insecurity occurrence experience questions with three possible answers, and a total of 27 scores. Then, HFSS was categorized as; “Food Insecure” (when a household scored ≤ 1) and “Food Secured” (when a household scored ≥ 2) [20].

The household wealth index was computed using indicators for urban residents. The wealth status was determined through principal component analysis (PCA). Then, a total of eight components with Eigenvalues > 1 were identified to measure the living standard of the household. Then, the eight components were used to produce a common factor score. Finally, the factor score was summed and ranked into tertiles as poor, medium and rich based on the lower, in the middle and higher score tertiles.

An anthropometric body measurement of height and weight were used to assess the nutritional status of participants. Height was measured to the nearest 0.1cm with a locally constructed portable wooden height. The participant's head was in the Frankfurt plane during measurement, knees were straight and the heels, buttocks and the shoulders blades had touched the vertical board. The weight was measured with a portable digital weight scale (Seca-Germany). Participants were informed to wear minimum clothing and stand still in the middle of the scale’s platform. Reading of weight was taken twice to the nearest 0.1 kg. A mathematical calculation of Body Mass Index (BMI) was used to determine the nutritional outcomes. The nutritional status was defined as CED when the BMI level of < 18.5kg/m^2^, good nutritional status (when BMI level was 18.5–24.9), overweight (when BMI of 25–29.9), and obese (when BMI of ≥ 30).

### Data quality control

To maintain the consistency of the data, the questionnaire was first translated from English to Amharic and retranslated back to the English language (S1 File). A single day of training was given to the recruited data collectors and supervisor about the aim of the study, ethical issues, anthropometric measurement, and data collection techniques. Before a week of the actual data collection period, the questionnaire was pre-tested among 5% of estimated sample size out of the study area. During the pre–test, the acceptability of the data collection process was evaluated. The entire questionnaire was checked for completeness, accuracy, and consistency by the supervisor and principal investigator in a day to day activity. All the data collection process was strongly coordinated and evaluated by the respective investigators through close supervision of data collectors to maintain the validity and reliability of the data.

### Data processing and analysis of the study

Data were coded and entered into Epi-info version7 software and exported to Statistical Package for Social Science (SPSS) version 20 for analysis. Frequency and cross tabulations were used to summarize descriptive statistics; and the result is presented using text and tables. A binary logistic regression model was fitted to identify factors associated with CED. Variables with a p-value of less than 0.2 in the bivariable analysis were fitted into the multivariable logistic regression analysis. Both crude odds ratio (COR) and adjusted odds ratio (AOR) with the corresponding 95% confidence interval (CI) was calculated to show the strength of association. Finally, a p-value of 0.05 was used to determine if the association was statistically significant. Model fitness was checked using Hosmer Lemishow test (p = 0.675).

### Ethics approval and consent to participate

The study protocol was approved by the Institutional Review Board (IRB) of the University of Gondar. All participants were informed about the purpose of the study, and the interview was held only with those who agreed to give verbal consent to participate in the study. The right of a participant to withdraw from the study at any time and without any precondition was disclosed clearly. Moreover, the confidentiality of information was guaranteed by using code numbers rather than personal identifiers and by keeping the questionnaire locked.

## Results

### Socio-demographic and economic related characteristics of study participants

A total of 892 people aged ≥ 65 years participated in this study. The mean (±SD) of the study participants was 69.85±6 years. Near to sixty percent, (59.1%), of the study participants were females. The mean (± SD) household family size was 4.68 (±1.86). The majority, (87.1%), of the respondents were Orthodox Christians in their religion. Regarding their marital status, more than fifty five percent, (56.7%), of the study participants were married. About 60.8% of respondents were unable to read and write. One third, (33.3%), of the study participants were poor in their living standard. Furthermore, one fourth, (25.7%), of the elders’ household was food in–secured (Table 1).

**Table 1:** Socio-demographic and economic characteristics of elders in Aykel town administration, Northwest Ethiopia, March 28^th^ to April 20^th^ 2018 (N = 892).

Variables	Frequency	Percentage
**Sex**		
Female	527	59.1
Male	365	40.9
**Age (in years)**		
65–74	732	82.1
75–84	120	13.4
≥ 85	40	4.5
Living arrangement		
With partner	506	56.7
With children	338	37.9
Alone	48	5.4
**Religion**		
Orthodox	777	87.1
Muslim	115	12.9
**Marital status**		
Married	506	56.7
Widowed	235	26.4
Divorced	143	16.0
Single	8	0.9
**Educational status**		
Unable to read and write	542	60.8
Able to read and write	227	25.4
Primary education	45	5.0
Secondary education	37	4.2
College and above	41	4.6
**Employment status**		
Housewife	375	42.0
Merchant	173	19.4
Daily labourer	136	15.3
Farmer	94	10.5
Governmental employed	65	7.3
Retired	49	5.5
**Household family size**		
≤ 3	207	23.2
≥ 4	676	76.8
**Household wealth index**		
Poor	297	33.3
Medium	296	32.2
Rich	299	33.5
**HFSS**		
Food secured	663	74.3
Food in–secured	229	25.7

### Co-morbidity, health care service and functional mobility related characteristics

About 33.5% of the respondents had a history of chronic illness during the interview. More than two-third, (67.7%), of the participant had a complaint of illness in the past 3 months before the interview. Concerning the types of chronic diseases, 47.92% had hypertension, 14.93% diabetic mellitus, 12.5% asthma and 9.72% HIV/AIDS. From the diseased respondents, 44.2% of the elderly had been suffered from declined food intake because of illness (Table 2).

**Table 2:** Co–morbidity, health care and functional mobility related characteristics of elders in Aykel town administration, Northwest Ethiopia, March 28^th^ to April 20^th^ 2018 (N = 892).

Variables	Frequency	Percentage
**History of Illness in the past 3 months**		
No	604	67.7
Yes	288	32.3
**History of chronic diseases**		
Yes	593	66.5
No	299	33.5
**Illness in the past 2 weeks of the survey**		
No	765	85.8
Yes	127	14.2
**Visit health facility (n = 362)**		
No	61	16.9
Yes	301	83.1
**Taking medication (n = 362)**		
No	56	15.5
Yes	306	84.5
**Number of medication (n = 362)**		
≤ 2	247	68.2
≥ 3	155	31.8
**Functional mobility status**		
No	10	1.1
Yes	882	98.9

### Dietary diversity and feeding practice related characteristics

The median DDS was 5.0. About 46.6%, 47.3% and 6.1% of the study participants had high, medium and low DDS, respectively. The mean (±SD) meal frequency of the study participants was 2.74 (± 0.59). Near to eighty percent, (79%), of the respondents had regular meal practice. More than 85.0% of the elderly ate their food together with their family. Almost all, (99.7%), of the elderly were able to feed themselves without any difficulty (Table 3).

**Table 3:** Feeding practices related characteristics of elders s in Aykel town administration, Northwest Ethiopia, March 28^th^ to April 20^th^ 2018 (N = 892).

Variables	Frequency	Percentage
**Meal frequency**		
≥ 3 times	506	56.7
≤ 2 times	386	43.3
**Time to have a meal**		
Regular	705	79.0
Irregular	187	21.0
**With whom you are feeding**		
With family	763	85.5
Alone	129	14.5
**Feeding ability of the respondent**		
By him/herself	889	97.7
With others support	3	0.3
**DDS**		
Low	54	6.1
Medium	422	47.3
High	416	46.6

### Prevalence of chronic energy deficiency

In the present study, the prevalence of CED was 17.6% (95%CI: 15.0, 20.20) among the study participants. Whereas, about 82.4% of the study participants had a BMI ≥ 18.5 kg/m^2^.

### Factors associated with chronic energy deficiency

In the bivariate logistic regression analysis, age, sex, marital status, illness in the past 3 months, history of chronic illness, visit health facility, taking medication, wealth index, dietary diversity score, food security status, loss of appetite and meal frequency had shown a significant association with CED. However, sex, age, dietary diversity score, food security status, wealth index and declined food intake due to illness were remained significantly associated with CED in the multivariable logistic regression analysis. Consequently, the odds of CED were 1.58 times higher among females than males (AOR: 1.58; 95%CI: 1.04, 2.41). As well, the likelihoods of CED were near to four times more likely among elders aged ≥ 85 years (AOR: 3.90; 95%CI: 1.85, 8.25). Likewise, the odds of CED was 1.95 times higher among those food insecured participants (AOR: 1.95; 95%CI: 1.26, 3.00). As compared to higher wealth status, those participants who had poor household wealth status were near to two times more likely to have the risk of CED (AOR: 1.77; 95%CI: 1.07, 2.94). Also, loss of appetite due to illness increased the odds of CED among participants (AOR: 2.93; 95%CI: 1.92,-4.48). Furthermore, the likelihoods of CED were more than five times higher among those elders who had poor DDS compared to their counterparts (AOR: 5.51; 95%CI: 2.89, 10.52) (Table 4).

**Table 4:** Factors associated with CED among elders in Aykel town administration, Northwest Ethiopia, March 28^th^ to April 20^th^ 2018 (N = 892).

Variables	CED		COR (95% CI)	AOR (95% CI)
	Yes (%)	No (%)		
**Sex**				
Female	111 (21.1)	416 (78.9)	1.85 (1.27–2.69)	1.58 (1.04–2.41)**
Male	46 (12.6)	319 (87.4)	1.00	1.00
**Age**				
65–74	115 (15.7)	617 (84.3)	1.00	1.00
75–84	28 (23.3)	92 (76.7)	1.65 (1.03–2.63)	1.45 (0.86–2.44)
≥ 85	14 (35.0)	26 (65.0)	3.25 (1.66–6.36)	3.90 (1.85–8.25)**
**HFSS**				
Food in-secured	67 (29.3)	162 (70.7%)	2.63 (1.84–3.78)	1.95 (1.26–3.00)**
Food secured	90 (13.6)	573 (86.4%)	1.00	1.00
**Household wealth index**				
Poor	80 (26.9)	217 (73.1)	2.53 (1.65–3.88)	1.77 (1.07–2.94)**
Medium	39 (13.2)	257 (86.8)	1.04 (0.65–1.68)	0.87 (0.52–1.47)
Rich	38 (12.7)	261(12.3)	1.00	1.00
**Meal frequency**				
≤ 2	85 (22.0)	302 (78)	1.69 (1.20–2.39)	1.43 (0.98–2.10)
≥ 3	72 (14.3)	433 (85.7)	1.00	1.00
**Loos of appetite due to illness**				
Yes	56 (35)	104 (65)	3.36 (2.29–4.95)	2.93 (1.92–4.48)**
No	101 (13.8)	631 (86.2)	1.00	1.00
**DDS**				
Low	25 (46.3)	29 (53.7)	6.31 (3.43–11.63)	5.51 (2.89–10.52)**
Medium	82 (19.4)	340 (80.6)	1.77 (1.21–2.59)	1.21 (0.80–1.83)
High	50 (12.0)	366 (88.0)	1.00	1.00

**Significant at p-value < 0.05, COR: crud odds ratio, AOR: adjusted odds ratio,

## Discussion

The overall prevalence of CED among older was 17.6% (95% CI: 15%, 20.2%) in the surveillance site. In addition, female sex, age, household food insecurity, poor wealth status and DDS were positively associted with CED.

The current finding of 17.6% CED was consistent with studies in Brazile18.8% [21]. However, the result was higher than reports in Sire Lanka 13.6% [22], South Africa 5.5% [23] and Iran 12% [24]. The possible reasons for this discrepancy among studies could be due to the difference in the economic power and the living standards of the participants. As compared to the previous studies, the present study was carried out among participants found in the less developed nation. This might result in lower food purchasing power of participants to diversified food items, and limit their dietary intakes. It is the fact that low dietary intake is the principal cause of CED. Another possible explanation could be due to the cultural and religious difference among participants.

In another way, the finding was lower than a similar local study reported in Northwest Ethiopia 21.9% [7]. The possible explanation might be due to the high proportion, (61.7%), of the study participants were widowed in the previous study. The study has been confirmed that widowed elders are found at higher risk of CED [12]. The possible explanation could be increased work load, lack of support and care may result in high prevalence of CED. Another possible reason might be the seasonal difference between studies. The present study was done in the post-harvest season whereas the study conducted at Gondar was in the pre-harvest season. Seasonal difference has an impact on food price, accessibility and availability of variety of food in the community. As a result low diversified, low intake of food will result in the high prevalence of chronic energy deficiency.

The odds of CED were more likely among female than male elders. This finding was congruent with reports in other parts of Ethiopia [7], Bangladesh [25], Helsinki [13], France [11] and Italy [26]. This could be because of that female elderly have low socio-economic conditions [27]; multiple pregnancies [28], high workload, cultural beliefs [29, 30], gender discriminations and less health seeking behavior, which negatively influence women’s health and nutritional status [25]. Moreover, the existing nutritional interventions in Ethiopia are not inclusive of all age group. The interventions are primarily focusing on women of child-bearing age, female adolescent and children. However, the result was contradicted by other studies done in Kenya [10], South Africa [31, 32] and Southern Brazil [21, 33]. The previous studies have been revealed that the likelihoods of CED were higher among male elders.

The odds of CED were more likely among elders aged ≥ 85 years than those 65–74 years. This finding was similar to studies conducted in Ethiopia [7], Sir Lanka [22], and France [11]. It might be due to the likely of combinative functional changes of elders’ such as; altered physiological or psychological problems, loss of sense of taste and smell, dental status and swallowing problems. This age related functional changes might impact the level of food intake, and result in poor health and nutritional outcomes. In addition, the gradual decline of physical capabilities, such as movement and mobility may be more likely among elders aged ≥ 85 years than their counterparts. This can lead to changes in food choice, eating habits and dietary intake, subsequently increasing the risk of CED.

The odds of being chronically energy deficient among older people were strongly associated with household food insecurity. The result was supported by a study done in the Sub Sahara African region [15, 34]. It could be due to that food insecurity may strongly limit household access to adequate quantity and quality of foods. Then, this could ultimately result in unhealthy nutritional outcomes among family members. The inability of household members to access adequate quantity and quality of foods is an important determinant of undernutrition. Moreover, it has been argued that household food insecurity is itself a form of material hardship [35], leading to feelings of deprivation, anxiety and poor mental health and possible social and behavioral difficulties. These all affect household members depending on their age and gender and result in a varied degree of poor nutritional outcomes [36].

Poor dietary diversity score practices of participants were another possible risk factor of CED. The result was in line with studies done in Ethiopia [7], and Sub-Sahara African region [15]. This is due to the fact that DDS is an important measure for individual’s diet quantity and quality, and thus poor DDS may result in poor nutritional outcomes. Moreover, DDS is associated with the economic ability of an individual to afford and access for varied types of food groups and better nutritonal status [37].

The household wealth index was another possible risk factor for elders’ CED. The result was supported by studies done in the USA [38], and France[11, 39]. Hence, CED was more likely among those elders who had poor household incomes. This was explained that household food purchasing power is totally depending on the level of incomes. As a result, the lower the living standard of the household might increase family members to have poor care and feeding of inadequate quantity and quality of foods. This result negatively to different levels of unhealthy nutritional outcomes in household members. Moreover, it was evidenced by the magnitude of undernutrition escalates obviously in that household with poor economic status [40].

In this study, loss of appetite due to illness was independently associated with elders’ CED. The previous study has been showed that poor appetite due to illness increases the risk of CED [41]. The previous study has been showed that poor appetite due to illness increases the risk of CED. The possible reason could be due to that because of illness is a pathological condition; it can strongly reduce individuals’ level of appetite for food and feeding. This might positively increase the risk of CED among elders. This was more evidence that a disease condition of an individual always causes loss of appetite, altered body nutrient metabolism, weakens the immune system and positively result in poor nutritional status [42, 43]. As well as, it is clear that illness is also the consequence of chronic undernutrition, whereby long-term nutrient deficiencies can impact the level of appetite.

Finally, the study tried to show the magnitude and determinants of CED in a well-defined population by using a larger sample size. However, some of the limitations of this study should be taken into consideration. Firstly, the study may not be totally free from measurement bias while conducting anthropometric assessments. Secondly, the measurement of participants feeding practice**s** and history of illness relied on memory, so there might be a chance to commit recall bias.

## Conclusion

The magnitude of CED was high among the study community. It was significantly associated with female sex, age, poor dietary diversity score, loss of appetite due to illness, household food insecurity and poor wealth status. Therefore, there is a need to design and implement programs and strategies to improve nutritional status particularly focusing on female older population in improving dietary practices and food security. In addition, improving household economic and living standards is an essential measure to address the burden of CED among the older community.

## Supporting information

S1 File English and Amharic version questionnaire. (PDF)

S2 File Data set used for analysis. (SAV)

## Abbreviations

AOR: Adjusted Odds Ratio
BMI: Body Mass Index
CI: Confidence Interval
COR: Crude Odds Ratio
DDSs: Dietary Diversity Scores
FANTA: Food and Nutrition Technical Assistance
HDSS: Health and Demographic Surveillance System
HFSS: Household Food Security Status
IRB: Institutional Review Board
NCD: Non-Communicable Disease
PCA: Principal Component Analysis
SD: Standard Deviation
WHO: World Health Organization
