# Supplementary material for: Chronic energy deficiency and associated factors among older population in Ethiopia: A community based study
Source: PLoS One. 2019 Apr 10;14(4):e0214861. doi: 10.1371/journal.pone.0214861 (PMC6457535; doi:10.1371/journal.pone.0214861)
Supplement: S1 File — (PDF) [file pone.0214861.s001.pdf]

English version questionnaire

Description	Identification
Region	Amhara regional state
Zone	Central Gondar
Woreda	Aykel town
Kebele	
Supervisor name and signature	
Data collector name and signature	
I.D number/House number	
Date of the interview	**______/______/2018**

1. Socio demographic and economic characteristics

S.no.	Questions	Response	skip
101	Sex of the respondent	1. Male [___]<br>2. Female [___]	
102	Age of the respondent (in years)	[____________]	
103	Religion	1. Orthodox [___]<br>2. Muslim [___]<br>3. Protestant [___]<br>4. Catholic [___]	
104	Marital status	1. Single [___]<br>2. Married [___]<br>3. Divorced [___]<br>4. Windowed [___]	
105	Educational status	1. unable to read and write [___]	

		2. able to read and write [___]<br>3. Primary school(grade1-8) [___]<br>4. Secondary school(grade 9-12) [__]<br>5. college and above [___]	
106	Partners' /care giver educational level	1. unable to read and write [___]<br>2. able to read and write [___]<br>3. Primary school(grade1-8) [___]<br>4. Secondary school(grade 9-12) [__]<br>5. college and above [___]	
107	Occupation of the respondent	1. Merchant [___]<br>2. Government employ [___]<br>3. Daily labor [___]<br>4. Housewife [___]<br>5. Farmer [___]<br>6. Other(specify) [___]	
108	Partner /care giver Occupation	1. Merchant [___]<br>2. Government employ [___]<br>3. Daily labor [___]<br>4. Housewife [___]<br>5. Farmer [___]<br>6. Other(specify) [___]	
109	With whom you are living?	1. With partner [___]<br>2. With Children's [___]<br>3. Lives alone [___]<br>4. Other (mention) [___]	
110	House hold family size?	[____________]	
111	Source of house hold food	1. From market [__] 2. From agriculture land [__] 3. other specify....................	

2 co-morbidities and Health related factors			
201	Have You ever ill for the last 3 months?	1. Yes [___]<br>2. No [___] →	203
202	If Q201 yes could you tell me the disease?	Specify.................................	
203	Do you have history of any chronic diseases?	1. Yes [___]<br>2. No [___] →	205
204	If Q203 yes, which one of the following (multiple answers are possible)	1. hypertension [___]<br>2. Diabetes Mellitus [___]<br>3. TB [___]<br>4. HIV/ADIS [___]<br>5. Other specify? [___]	
205	Did you face any illness in the past 2 weeks?	1. Yes [___]<br>2. No [___] →	207
206	If Q205 is yes what was the illness?	1. Diarrhea [___]<br>2. Nausea [___]<br>3. Vomiting [___]<br>4. Other specify?..................	
207	Have you Visit health facility?	1. Yes [___]<br>2. No [___]	
208	Have you taking medication?	1. Yes [___]<br>2. No [___] →	210
209	Number of medication you are taking mention?	[______________]	
210	Functional mobility	1. Able to move [__]<br>2. unable to move [__]<br>3. With the help of others [__]<br>4. Other specify [__]	

3	**Feeding practices**		
301	Have you decline food intake because of illness?	1. Yes [___]<br>2. No [___]	
302	Meal frequency practice in the previous 24 hours	[___________]times	
303	Ability of the respondent to feed?	1. By him/herself [__]<br>2. With the help of others [__]	
304	With whom you are feeding?	1. Always alone [__]<br>2. Sometimes alone [__]<br>3. Always with family Members [__]<br>4.Sometimes with family members [__]	
305	Time to have meal?	1. Regular (at the time of breakfast, lunch, snack and dinner) [__]<br>2. Irregular (any time s/he eat when they get food) [__]	

Breakfast	Lunch	Snack	Dinner	

Dietary information

Fill the table based on food group by referring the information Recorded above, if individual ate at least one item score 1 and if they didn't ate anything score 0

	Food group	Items	Yes=1<br>No=0
1	Cereals	Teff, Wheat, Barley, Maize, Rice, corn/maize, sorghum, millet or any other grains or foods made from these(e.g. bread, porridge or other grain products)	

2	Vitamin A rich vegetables and fruits	Carrots, sweet potatoes, orange, avocado, mangoes, papaya, lemon.	
3	White roots and Tubers	white potatoes, yams	
4	Dark green leafy vegetables;	Spinach, avocado, endive, Swiss chard, salad, pepper	
5	Other vegetables, fruits	Tomato, Onion, garlic, banana ,other wild fruits, juice	
6	Organ and flesh	liver, kidney, other organ meats or blood-based foods	
7	Eggs	Raw eggs, roasted eggs	
8	Legumes, nuts and seeds	beans, peas, lentils, nuts, seeds or foods made from these	
9	Milk and milk Products	milk, cheese, yogurt or other milk products	
10	Oils and fats	oil, fats or butter added to food or used for cooking	
11	Sweet	sugar, honey, sweetened soda or sugary foods such as chocolates, candies, cookies and cakes	
12	Spices, condiments, Beverages	spices(black pepper, salt), condiments (soy sauce, coffee, tea, alcoholic beverages ,chilies, local beers	
Individual level only		Did you eat anything (meal or snack) OUTSIDE of the home yesterday?	

3 Wealth index characteristics

Code	Questions	Response	Skip

401	Owner ship of the house	1. Private<br>2. Rented from individual<br>3. kebele<br>4. Other(specify)___________	
402	Number of rooms	____________in number	
403	Main material of the dwelling floor	1. Earth / Soil<br>2. Dung<br>3. Cement<br>4. Ceramics<br>5. Carpet<br>6. Others (specify)__________	
404	Main material of the roof	1. Iron corrugated sheet<br>2. Thatch<br>3. Others (specify)__________	
405	Main material of the exterior walls	1. Stone with mud<br>2. Wood with mud<br>3. Stone with cement<br>4. Others (specify)__________	
406	Type of fuel mainly used for household cooking	1. Electricity<br>2. Charcoal<br>3. Wood<br>4. Animal dung<br>5. Others(specify)___________	
407	Is the cooking usually done in the house, in a separate building, or outdoors?	1. In a separate room used as kitchen<br>2. Elsewhere in the house<br>3. In a separate building<br>4. Outdoors<br>5. Other (*specify*)___________	

408	Does any member of the household own any land that can be used for agriculture?	1. Yes<br>2. No →	411
409	Ownership of the farm land (if the household doesn't have one of the two option, use 999)	1. Own, in hectares/gemed____<br>2. Rent, in hectares/gemed____	
410	Annual total agricultural products (includes all items)	_______________kuintal	
411	Does your household have<br>A. Electricity?<br>B. A Radio?<br>C. A Television?<br>D. A Non-mobile telephone?<br>E. A Refrigerator?<br>F. Table?<br>G. Chair?<br>H. A bed with cotton/spring mattress	Yes No<br>1 0<br>1 0<br>1 0<br>1 0<br>1 0<br>1 0<br>1 0<br>1 0	
412	Does any member of your household own<br>A. A watch?<br>B. A mobile phone?<br>C. A bicycle?	Yes No<br>1 0<br>1 0	

	A. A Bajaj?<br>B. Animal drawn cart?<br>C. Car?	1 0<br>1 0<br>1 0<br>1 0	
413	Does this household own any livestock, herds, other farm animals, or poultry?	1. Yes<br>2. No →	415
414	How many of the following animals does the household have? (if the household does not have the listed animal use **999**)		
	A. Cattle, milk cows, bulls?	__________in number	
	B. Horses, Donkeys, or mules?	__________in number	
	C. Goats?	__________in number	
	D. Sheep?	__________in number	
	E. Chickens?	__________in number	
	F. Beehives?	__________in number	
415	Does any member of this household have a bank account?	1. Yes<br>2. No	

5 Household Food Insecurity Access Scale (HFIAS) for Measurement of Food Access

Household Food Insecurity Access Scale (HFIAS) Measurement Tool

No.	Question	Response Options	Code
501.	In the past four weeks, did you worry that your household would not have enough food?	0 = No (skip to Q2)<br>1=Yes	[____]
501.a	How often did this happen?	1 = Rarely (once or twice in the past four weeks)<br>2 = Sometimes (three to ten times in the past four weeks<br>3 = Often (more than ten times in the past four weeks)	[____]
502.	In the past four weeks, were you or any household member not able to eat the kinds of foods you preferred because of a lack of resources?	0 = No (skip to Q3)<br>1=Yes	[____]
502.a	How often did this happen?	1 = Rarely (once or twice in the past four weeks)<br>2 = Sometimes (three to ten times in the past four weeks<br>3 = Often (more than ten times in the past four weeks)	[____]
503.	In the past four weeks, did you or any household member have to eat	0 = No (skip to Q4)<br>1 = Yes	

	a limited variety of foods due to a lack of resources?		
503.a	How often did this happen?	1 = Rarely (once or twice in the past four weeks)<br>2 = Sometimes (three to ten times in the past four weeks<br>3 = Often (more than ten times in the past four weeks)	[____]
504.	In the past four weeks, did you or any household member have to eat some foods that you really did not want to eat because of a lack of resources to obtain other types of food?	0 = No (skip to Q5)<br>1 = Yes	
504.a	How often did this happen?	1 = Rarely (once or twice in the past four weeks)<br>2 = Sometimes (three to ten times in the past four weeks<br>3 = Often (more than ten times in the past four weeks)	[____]
505.	In the past four weeks, did you or any household member have to eat a smaller meal than you felt you needed because there was not enough food?	0 = No (skip to Q2)<br>1=Yes	[____]

505.a	How often did this happen?	1 = Rarely (once or twice in the past four weeks)<br><br>2 = Sometimes (three to ten times in the past four weeks<br><br>3 = Often (more than ten times in the past four weeks)	[____]
506.	In the past four weeks, did you or any other household member have to eat fewer meals in a day because there was not enough food?	0 = No (skip to Q2)<br><br>1=Yes	[____]
506.a	How often did this happen?	1 = Rarely (once or twice in the past four weeks)<br><br>2 = Sometimes (three to ten times in the past four weeks<br><br>3 = Often (more than ten times in the past four weeks)	[____]
507.	In the past four weeks, was there ever no food to eat of any kind in your household because of lack of resources to get food?	0 = No (skip to Q2)<br><br>1=Yes	[____]
507.a	How often did this happen?	1 = Rarely (once or twice in the past four weeks)<br><br>2 = Sometimes (three to ten times in the past four weeks	[____]

		3 = Often (more than ten times in the past four weeks)	
508.	In the past four weeks, did you or any household member go to sleep at night hungry because there was not enough food?	0 = No (skip to Q2)<br><br>1=Yes	[____]
508.a	How often did this happen?	1 = Rarely (once or twice in the past four weeks)<br><br>2 = Sometimes (three to ten times in the past four weeks<br><br>3 = Often (more than ten times in the past four weeks)	[____]
509.	In the past four weeks, did you or any household member go a whole day and night without eating anything because there was not enough food?	0 = No (skip to Q2)<br><br>1=Yes	[____]
509.a	How often did this happen?	1 = Rarely (once or twice in the past four weeks)<br><br>2 = Sometimes (three to ten times in the past four weeks<br><br>3 = Often (more than ten times in the past four weeks)	[____]

6 Anthropometric body measurement characteristics

S. No	Anthropometric body measurements	Measurements		
		1	2	Average
601	Weight of the participant in kilo gram(Kg)	[___]Kg	[___]Kg	[___]Kg
602	Height of the participant in meter (m)	[___]m	[___]m	[___]m

Amharic version questionnaire

መግለጫ	መለያ
ክልል	አማራ ክልላዊ መንግስት
ዞን	ማዕከላዊ ጎንደር
ወረዳ	አይከል ከተማ አስተዳደር
ቀበሌ	
የሱፐርቫይዘር ስምና ፊርማ	
የመረጃ ሰብሳቢው ስምና ፊርማ	
የተሳታፊ መለያ ኮድ	
ቀን	______/______/2018

**1. ማህበራዊ ፣ ስነ- ህዝባዊ እና ኢኮኖሚያዊ ጥያቄዎች**			**ወደ ሚቀጥለው ተሻገር**
ተ.ቁ ጥር	ጥያቄዎች	የመልስ አማራጮች	
101	የተሳታፊ ፆታ	1. ወንድ [___]<br>2. ሴት [___]	
102	የተሳታፊ እድሜ	[_______________]	
103	ሐይማኖት	1. ኦርቶዶክስ [___]<br>2. ሙስሊም [___]<br>3. ፕሮቴስታንት [___]<br>4. ካቶሊክ [___]	
104	የትዳር ሁኔታ	1. ያላገባ/ች [___]	

		2. ያገባ/ች [___]<br>3. የፈታ/ች [___]<br>4. የሞተችበት/ባት [___]	
105	የትምህርት ደረጃ	1. መፃፍ ማንበብ የማይችል/የማትችል [___]<br>2. መፃፍ ማንበብ የሚችል/የምትችል [___]<br>3. 1ኛ ደረጃ/1-8 ክፍል/ [___]<br>4. 2ኛ ደረጃ/9- 12 / [___]<br>5. ዲፕሎማና ከዚያ በላይ [___]	
106	የትዳር አጋር የትምህርት ደረጃ	1. መፃፍ ማንበብ የማይችል/የማትችል [___]<br>2. መፃፍ ማንበብ የሚችል/የምትችል [___]<br>3. 1ኛ ደረጃ/1-8 ክፍል/ [___]<br>4. 2ኛ ደረጃ/9- 12 / [___]<br>5. ዲፕሎማና ከዚያ በላይ [___]	
107	የስራ ሁኔታ	1. ነጋዴ [___]<br>2. የመንግስት ሰራተኛ [___]<br>3. የቀን ሰራተኛ [___]<br>4. የቤት እመቤት [___]<br>5. ገበሬ [___]<br>6. ሌላ [___]	
108	የትዳር አጋርዎ የስራ ሁኔታ	1. ነጋዴ [___]<br>2. የመንግስት ሰራተኛ [___]<br>3. የቀን ሰራተኛ [___]<br>4. የቤት እመቤት [___]<br>5. ገበሬ [___]<br>6. ሌላ/ይገለፅ/ [___]	
109	ከማን ጋር ነው የሚኖሩት?	1. ከትዳር አጋርዎ ጋር [___]	

		2. ከልጆችዎ ጋር [___]<br>3. ብቻዎትን [___]<br>4. ሌላ (ይጠቀስ) [__________]	
110	የቤተሰብ ብዛት	[__________]	
111	የምግብ እህል የምታገኙት ከየት ነው?	1. ከገበያ 2. ከእርሻ መሬት 3. ሌላ ይገለፅ..................	
**2. የጤና ሁኔታ**			
201	ባለፉት ሶስት ወራት ውስጥ ታመው ያውቃሉ?	1. አዎ [___]<br>2. አላመመኝም [___]	
202	ለጥ.ቁ. 201 መልስዎ አዎ ከሆነ ያመመዎ ምንድን ነበር?	ይጥቀሱ......................	
203	ከዚህ በፊት ስር የሰደደ(የቆየ) በሽታ እንዳለብዎ ተነግሮዎት ያውቃል?	1. አዎ [___]<br>2. አላመመኝም [___]	
204	ለጥ.ቁ. 203 መልስዎ አዎ ከሆነ ያመመዎ የትኛው በሽታ ነው?	1. ደም ግፊት [___]<br>2. ስኳር [___]<br>3. ቲቢ [___]<br>4. ኤች. አይ.ቪ [___]<br>5. ሌላ (ይገለፅ) [________________]	
205	ባለፉት ሁለት ሳምንት ውስጥ ታመው ያውቃሉ?	1. አዎ [___]<br>2. አላመመኝም [___]	
206	ለጥ.ቁ. 203 መልስዎ አዎ ከሆነ ያመመዎ የትኛው በሽታ ነው?	1. ተቅማጥ [___]<br>2. ማቅለሽለሽ [___]<br>3. ትውኪያ [___]	

		4. ሌላ (ይገለፅ) [____________________]	
207	ወደ ጤና ተቋም ሄደው ነበር ?	1. አዎ [___]<br>2. አልሄድኩም [___]	
208	ለህመመዎ መድሃኒት ወስደዋል?	1. አዎ [___]<br>2. አልዎሰድኩም [___]	
209	የሚወስዱት መድሀኒት ብዛት ስንት ነው?	[______________________]	
210	የመንቀሳቀስ ችሎታዎ በተመለከተ ምን ይመስላል?	1. በራስ ችሎ መንቀሳቀስ የሚችል/የምትችል [__]<br>2. በሌላ ሰው ድጋፍ የሚንቀሳቀስ/የምትንቀሳቀስ [__]	
**3**	**የአመጋገብ ልማድ**		
301	በህመም ምክንያት የምግብ ፍላጎትህ ቀንሷል?	1. አዎ [___]<br>2. አልቀነሰም [___]	
302	ትናንት (በ24 ሰዓት ውስጥ) ስንት ጊዜ ተመግበሃል?	1. አንድ ጊዜ [___]<br>2. ሁለት ጊዜ [___]<br>3. ሶስት ጊዜ [___]<br>4. አራት ጊዜ [___]<br>5.አምስትና ከዚያ በላይ [___]	
303	ራስን የመመገብ ችሎታ?	1. በራሱ/ሷ መመገብ የሚችል/የምትችል [___]<br>2. በሌላ ሰው ድጋፍ የሚመገብ/የምትመገብ [___]<br>3. ሌላ ይገለፅ[___]	

304	ማዕደዎን ከማን ጋር ነው የሚመገቡት ?	1. ሁልጊዜ ብቻዎትን [___]<br>2. አንዳንድጊዜ ብቻዎትን [___]<br>3. ሁልጊዜ ከቤተሰብ ጋር [___]<br>4. አንዳንድጊዜ ከቤተሰብ ጋር [___]	
305	የማዕድ አመጋገብ ጊዜዎ ምን ይመስላል?	1. ሰዓቱን ጠብቆ ቁርስ፣ ምሳ፣ መክሰስ እና እራት [___]<br>2. በተገኘው ጊዜ ከመመገቢያ ሰዓት ውጭ [___]<br>3. ሌላ ይገለፅ [___]	

ባለፈዉ ቀን እና ማታ በ24 ሰአት ዉሰጥ የበላሀዉን/ሽዉን ከጠዋት ጀምሮ ለጠያቂዉ ተናገሪ/ር

ቁርስ	ምሳ	መክሰስ	ራት

ከላይ ሰንጠረዡን በማየት በ24 ሰአት የተመገቡትን ከምግብ ዝርዝሩ 1 እና ከዛ በላይ ከበላ/ላች 1 ይሙሉ ፤ከምግብ ዝርዝሩ ምንም አይነት ምግብ ካልተመገበ/ች 0 ይሙሉ።

	የምግብ ምድብ	ዝርዝሮች	አዎ=1<br>የለም=0
1	እህልና የእህል ዘሮች	ጤፍ፣ ስንዴ፤ ገብስ፣ ማሽላ፣ በቆሎ፣ ሩዝ፣ ዳጉሳ (ዳቦ፣ እንጀራ፤ ንፍሮ፤ ቆሎ፤ ገንፎ፣ ፓስታ፤ መኮረኒ)	
2	በቫይታሚን ኤ የበለጸጉ ፍራፍሬ<br>እና ስራ ስር ምንጮች	ስኳር ድንች፣ ፓፓያ፤ ማንጎ፤ ካሮት፤ ትርንጎ፤ አቮካዶ፤ ብርቱካን፤ ሎሚ	
3	ስራ ስሮች	ቀይ ስር፤ ድንች	

4	ጥቁር አረንጓዴ ቅጠል ያላቸዉ የጓሮ አትክልቶች	ሰላጣ፤ ቆስጣ፤ ጎመን፤ ቃሪያ፤ አቮካዶ፤ ጥቅል ጎመን	
5	ሌሎችየጓሮ አትክልቶች እና ፍራፍሬዎች	ቲማቲም፣ ቀይ ሽንኩርት፤ ነጭ ሽንኩርት፤ ዋንዛ፤ እንጆሪ፤ ሙዝ፤ የአትክልት ጭማቂዎች	
6	ስጋ እና የስጋ ዉጤቶች	ኩላሊት፤ ጉበት፤ ጥብስ፤ ቅቅል፤ ዶሮ ወጥ፤ ቀይ ወጥ	
7	እንቁላል	እንቁላል ፍርፍር፣ እንቁላል በስጋ፤ እንቁላል ሳንዱች	
8	ጥራ ጥሬ	ባቄላ፣ አተር፣ ምስር፤ ዓያ፤ ሽንብራ፤ (ሽሮ ወጥ፤ ክክ፤ ምስር ወጥ)	
9	ወተት እና የወተት ምርቶች	ጥሬ ወተት፣ አይብ፣ እርጎ፤ አጓት	
10	ዘይት እና ስብ	ዘይት፣ ቅቤ፣ የአትክልት ቅቤ፣	
11	ጣፋጭ ምግቦች	ሰኳር፣ ማር፣ ለስላሳ መጠጦች፣ ቸኮሌት፣ ከረሚላ፤ ኩኪስ	
12	መጠጦች፤ቅመሞች	በርበሬ፣ ጨዉ፣ ቡና፣ ሻይ፣ የአልኮል መጠጦች	

4. የቤተሰብ የሃብት ምጣኔ ሁኔታ እና ተያያዥ ጥያቄዎች / ለከተማ ነዋሪዎች ብቻ

ተ. ቁ	ጥያቄ	የመልስ አማራጭ	
401	መኖሪያ ቤትዎ የማን ነው?	1. የእርስዎ(የግለዎ) [__]<br>2. ከግለሰብ ኪራይ [__]<br>3. ሌላ ይገለፅ........................	
402	ስንት ክፍሎች አሉት	...................በቁጥር	
403	የመኖሪያ ቤቱ ወለል ከምን የተሰራ ነው?	1. አፈር/አሸዋ [___]<br>2. በእበት የተለቀለቀ [___]<br>3. በስሚንቶ<br>4. ሸንበቆ<br>5. ስጋጃ/ምንጣፍ<br>6. ሌላ-----------------------	

404	የቤቱ ጣራ የተሰራው ከምንድን ነው?	1.ቀርቆሮ ክዳን [__]<br>2. እንጨት [__]<br>3. ሣር ክዳን [__]<br>4. ሸንበቆ [__]<br>5. ሌላ ---------------------	
405	የቤቱ ግድግዳ የተሰራው ከምንድን ነው?	1.ድንጋይ በጭቃ [___]<br>2. እንጨት በጭቃ [___]<br>3. ድንጋይ በስሚንቶ [___]<br>4. ሌላ ----------------------	
406	ምግብ ለማብሰል የምትጠቀሙት ምንድን ነው?	1.ኤሌክትሪክ [__]<br>2. ክሰል [__]<br>3. እንጨት [__]<br>4. ኩበት [__]<br>5. ሌላ ይገለፅ...........................	
407	ምግብ የሚበሰለው በተለምዶ ቤት ውስጥ ነው፣ በጢስ ቤት ነው ወይስ ከቤት ውጭ?	1. ቤት ውስጥ ራሱን በቻለ ክፍል[__]<br>2. ቤት ውስጥ በማንኛውም ቦታ[__]<br>3. ከቤቱ ውጭ በሆነ ጭስ ቤት [__]<br>4. ውጭ ላይ[__]<br>5. ሌላ ይገለፅ?...............................	
408	ከቤተሰብዎ አባል ውስጥ የእርሻ መሬት/ቦታ ያለው አለ?	1. አዎ [__]<br>2. የለም [__] →	411
409	የእርሻ መሬቱ ባለቤት ማን ነው?	1. የእርስዎ ከሆነ በሄክታር/በገመድ .....<br>2. የኪራይ ከሆነ በሄክታር/በገመድ.......	
410	በአመት ከሁሉም የእህል አይነት በኩንታል ስንት ያገኛሉ?	...............ኩንታል	
411	በቤትዎ ውስጥ ያሉ ቁሶች		
	ሀ. ኤሌክትሪክ አላችሁ?	ሀ. አዎን [__] ለ. የለም [__]	
	ለ. ሬድዮ አላችሁ?	ሀ. አዎን [__] ለ. የለም [__]	
	ሐ. ቴሌቭዥን አላችሁ?	ሀ. አዎን [__] ለ. የለም [__]	
	መ. የቤት ስልክ አላችሁ ?	ሀ. አዎን [__] ለ. የለም [__]	
	ሠ. ፍሪጅ አላችሁ?	ሀ. አዎን [__] ለ. የለም [__]	
	ረ. ሶፋ/ ወንበር አላችሁ?	ሀ. አዎን [__] ለ. የለም [__]	

	ሰ. ጠረጴዛ አላችሁ ?	ሀ. አዎን [__] ለ. የለም [__]	
	ሸ. አልጋ እና ከጥጥ /እስፓንጅ/ እስፕርንግ የተሰራ ፍራሽ/አላችሁ?	ሀ. አዎን [__] ለ. የለም [__]	
412	ከቤተሰብዎ አባል ውስጥ የሚከተሉትን ቁሶች ያለው አለ?		
	ቀ. የእጅ ሰዓት ?	ሀ. አዎን [__] ለ. የለም [__]	
	በ. ሞባይል?	ሀ. አዎን [__] ለ. የለም [__]	
	ተ. ሳይክል?	ሀ. አዎን [__] ለ. የለም [__]	
	ቸ. ባጃጅ?	ሀ. አዎን [__] ለ. የለም [__]	
	ነ. ጋሪ?	ሀ. አዎን [__] ለ. የለም [__]	
	ኘ. መኪና?	ሀ. አዎን [__] ለ. የለም [__]	
413	ላም፣ በሬ ፣ፍየል፣ ዶሮ አላችሁ?	ሀ. አዎን [__] ለ. የለም [__] →	415
414	ስንት የቁምና የጋማ ከብቶች አላችሁ ?		
	ሀ. ከብቶች, የወተት ላም, ኮርማ?	...........................በቁጥር	
	ለ. ፈረስ, አህያ, ወይም በቅሎ?	...........................በቁጥር	
	ሐ. ፍየል?	...........................በቁጥር	
	መ. በግ?	...........................በቁጥር	
	ሠ. ዶሮ?	...........................በቁጥር	
	ረ. የንብ ቀፎ?	...........................በቁጥር	
415	የባንክ ደብተር አላችሁ?	ሀ. አዎን [__] ለ. የለም [__]	

ክፍል 5 ፡- የቤተሰብ የምግብ ዋስትናን የሚዳስሱ ጥያቄዎች

ተ.ቁ	ጥያቄ	ምርጫ	መልስ
501	ባለፉት አራት ሳምንታት ውስጥ ቤተሰብዎ በቂ ምግብ አላገኘም ብለው አስበው (ተጨንቀው) ነበር?	ሀ/ አዎ<br>ለ/ የለም	[____]

501.ሀ	መልስዎ አዎ ከሆነ፣ ምን ያህል ጊዜ ተከስቶ ያዉቃል?	1 =አልፎ አልፎ (1 ወይም 2 ጊዜ)<br>2 =አንዳንድ ጊዜ (ከ 3-10 ጊዜ)<br>3 =ብዙ ጊዜ (ከ 10 ጊዜ በላይ)	[____]
502	ባለፉት አራት ሳምንታት ውስጥ እርስዎ ወይም ከቤተሰብ አባልዎ መካከል በገንዘብ እጥረት ምክንያት የመረጣችሁትን ምግብ ሳትመገቡ የቀራችሁበት ጊዜ ነበር	ሀ/ አዎ<br>ለ/ የለም	[____]
502.ሀ	መልስዎ አዎ ከሆነ፣ ምን ያህል ጊዜ ተከስቶ ያዉቃል?	1=አልፎ አልፎ (1 ወይም 2 ጊዜ)<br>2 =አንዳንድ ጊዜ (ከ 3-10 ጊዜ)<br>3 =ብዙ ጊዜ (ከ 10 ጊዜ በላይ)	[____]
503	ባለፉት አራት ሳምንታት ውስጥ እርስዎ ወይም ከቤተሰብ አባልዎ መካከል በገንዘብ እጥረት ምክንያት ውስን የምግብ አይነትተመግበዋል?	ሀ/ አዎ<br>ለ/ የለም	[____]
503.ሀ	መልስዎ አዎ ከሆነ፣ ምን ያህል ጊዜ ተከስቶ ያዉቃል?	1 =አልፎ አልፎ (1 ወይም 2 ጊዜ)<br>2 =አንዳንድ ጊዜ (ከ 3-10 ጊዜ)<br>3 =ብዙ ጊዜ (ከ 10 ጊዜ በላይ)	[____]
504	ባለፉት አራት ሳምንታት ውስጥ እርስዎ ወይም ከቤተሰብ አባልዎ መካከል በገንዘብ እጥረት ምክንያት ሌሎች የምግብ አይነቶችን ባለማግኘትዎ መመገብ የማይፈልጉትን የምግብ አይነት ተመግበዋል?	ሀ/ አዎ<br>ለ/ የለም	[____]
504.ሀ	መልስዎ አዎ ከሆነ፣ ምን ያህል ጊዜ ተከስቶ ያዉቃል?	1=አልፎ አልፎ (1 ወይም 2 ጊዜ)<br>2 =አንዳንድ ጊዜ (ከ 3-10 ጊዜ)<br>3 =ብዙ ጊዜ (ከ 10 ጊዜ በላይ)	[____]
505	ባለፉት አራት ሳምንታት ውስጥ እርስዎ ወይም ከቤተሰብ አባልዎ መካከል በገንዘብ እጥረት ምክንያት	ሀ/ አዎ<br>ለ/ የለም	[____]

	በቂ ምግብ ባለመኖሩ ከሚያስፈልግዎ (ከበቂ) በታች የሆነ ምግብ ተመግበው ያውቃሉ		
505.ሀ	መልስዎ አዎ ከሆነ፣ ምን ያህል ጊዜ ተከስቶ ያዉቃል?	1=አልፎ አልፎ (1 ወይም 2 ጊዜ)<br>2 =አንዳንድ ጊዜ (ከ 3-10 ጊዜ)<br>3 =ብዙ ጊዜ (ከ 10 ጊዜ በላይ)	[____]
506	ባለፉት አራት ሳምንታት ውስጥ እርስዎ ወይም ከቤተሰብ አባልዎ መካከል በገንዘብ እጥረት ምክንያት በቂ ምግብ ባለመኖሩ በቀን ውስጥ ከሚያስፈልገዎ ጊዜ በታች ተመግበው ያውቃሉ	ሀ/ አዎ<br>ለ/ የለም	[____]
506.ሀ	መልስዎ አዎ ከሆነ፣ ምን ያህል ጊዜ ተከስቶ ያዉቃል?	1=አልፎ አልፎ (1 ወይም 2 ጊዜ)<br>2 =አንዳንድ ጊዜ (ከ 3-10 ጊዜ)<br>3 =ብዙ ጊዜ (ከ 10 ጊዜ በላይ)	[____]
507	ባለፉት አራት ሳምንታት በቤተሰብዎ ውስጥ በገንዘብ እጥረት ምክንያት ምግብ ባለማግኘትዎ በቤትዎ ውስጥ ምንም አይነት የሚበላ ምግብ አጥተው ነበር	ሀ/ አዎ<br>ለ/ የለም	[____]
507.ሀ	መልስዎ አዎ ከሆነ፣ ምን ያህል ጊዜ ተከስቶ ያዉቃል?	1=አልፎ አልፎ (1 ወይም 2 ጊዜ)<br>2 =አንዳንድ ጊዜ (ከ 3-10 ጊዜ)<br>3 =ብዙ ጊዜ (ከ 10 ጊዜ በላይ)	[____]
508	ባለፉት አራት ሳምንታት ውስጥ እርስዎ ወይም ከቤተሰብ አባልዎ መካከል በገንዘብ እጥረት ምክንያት ምግብ ሳትመገቡ አድራችሁ ታውቃላችሁ	ሀ/ አዎ<br>ለ/ የለም	[____]
508.ሀ	መልስዎ አዎ ከሆነ፣ ምን ያህል ጊዜ ተከስቶ ያዉቃል?	1=አልፎ አልፎ (1 ወይም 2 ጊዜ)<br>2 =አንዳንድ ጊዜ (ከ 3-10 ጊዜ)<br>3 =ብዙ ጊዜ (ከ 10 ጊዜ በላይ)	[____]

509	ባለፉት አራት ሳምንታት ውስጥ እርስዎ ወይም ከቤተሰብ አባልዎ መካከል በገንዘብ እጥረት ምክንያት ሙሉ ቀንና ማታ ያለምንም ምግብ መመገብ ያሳለፋችሁት ጊዜ ነበር?	ሀ/ አዎ<br>ለ/ የለም	[____]
509.ሀ	መልስዎ አዎ ከሆነ፣ ምን ያህል ጊዜ ተከስቶ ያዉቃል?	1=አልፎ አልፎ (1 ወይም 2 ጊዜ)<br>2 =አንዳንድ ጊዜ (ከ 3-10 ጊዜ)<br>3 =ብዙ ጊዜ (ከ 10 ጊዜ በላይ)	[____]

6. አንትሮፖሜትሪክ የሰውነት ልኬት ሁኔታ

ተ.ቁ	**አንትሮፖሜትሪክ የሰውነት ልኬት**	ልኬቶች			
		ልኬት 1	ልኬት 2	አማካይ	
601	የተሳታፊ ክብደት በኪሎ ግራም	[____]ኪ.ግ	[____]ኪ.ግ	[____]ኪ.ግ	
602	የተሳታፊ ቁመት በሜትር	[___]ሜትር	[___]ሜትር	[___]ሜትር	
